# Topical Anti-Nuclear Factor-Kappa B Small Interfering RNA with Functional Peptides Containing Sericin-Based Hydrogel for Atopic Dermatitis

**DOI:** 10.3390/pharmaceutics7030294

**Published:** 2015-09-07

**Authors:** Takanori Kanazawa, Yuki Shizawa, Mayu Takeuchi, Kuniko Tamano, Hisako Ibaraki, Yasuo Seta, Yuuki Takashima, Hiroaki Okada

**Affiliations:** Department of Pharmaceutical Science, School of Pharmacy, Tokyo University of Pharmacy and Life Sciences, 1432-1 Horinouchi, Hachioji, Tokyo 192-0392, Japan; E-Mails: ykshizawa@gmail.com (Y.S.); mayu.2.takeuchi@gmail.com (M.T.); tamano.kuniko.f8@gmail.com (K.T.); ibaraki@toyaku.ac.jp (H.I.); setayas@toyaku.ac.jp (Y.S.); takasima@toyaku.ac.jp (Y.T.); okada@toyaku.ac.jp (H.O.)

**Keywords:** atopic dermatitis, skin topical application, siRNAs, NF-κB, sericin, hydrogel

## Abstract

The small interfering RNA (siRNA) is suggested to offer a novel means of treating atopic dermatitis (AD) because it allows the specific silencing of genes related to AD pathogenesis. In our previous study, we found that siRNA targeted against RelA, an important nuclear factor-kappa B (NF-κB) subdomain, with functional peptides, showed therapeutic effects in a mouse model of AD. In the present study, to develop a topical skin application against AD, we prepared a hydrogel containing anti-RelA siRNA and functional peptides and determined the intradermal permeation and the anti-AD effects in an AD mouse model. We selected the silk protein, sericin (SC), which is a versatile biocompatible biomaterial to prepare hydrogel as an aqueous gel base. We found that the siRNA was more widely delivered to the site of application in AD-induced ear skin of mice after topical application via the hydrogel containing functional peptides than via the preparation without functional peptides. In addition, the ear thickness and clinical skin severity of the AD-induced mice treated with hydrogel containing anti-RelA siRNA with functional peptides improved more than that of mice treated with the preparation formulated with negative siRNA.

## 1. Introduction

Atopic dermatitis (AD) is a chronic inflammatory disease of the skin with a complex pathogenesis that is associated with both genetic and environmental factors [1]. In its most severe form, AD results in a very low quality of life for the patient and, therefore, an efficacious, nontoxic medicinal ointment, or gel formulation for convenient topical application, is desirable.

The use of topical corticosteroids in combination with aggressive moisturizing is the most common and effective treatment for AD. However, the use of high-strength corticosteroids combined with an occlusive dressing can cause side effects, such as an increase in the risk of skin thinning. In addition, the long-term use of steroids can result in complications such as weight gain, reduced bone density, and suppression of the immune system [2].

Nuclear factor-kappa B (NF-κB) plays a critical role in the immunological disturbances observed in AD, and NF-κB decoy oligodeoxynucleotides (ODNs) have been reported to ameliorate atopic skin lesions in NC/Nga mice with fewer side effects than corticosteroids [3,4,5].

Small interfering RNA (siRNA) can specifically silence genes related to AD pathogenesis through a process called RNA interference (RNAi). Therefore, it is also expected to offer a potential means of treating AD without causing side effects. In our previous study, we reported several findings on the effects of treating AD with topical siRNAs targeted against RelA, which is the most important NF-κB subdomain involved in the production of inflammatory cytokines, as shown in the AD-induced mouse model [6]. We also found that the use of functional peptides, such as cell-penetrating or tight junction opening peptides to deliver the anti-RelA siRNA or RNAi reagents, was an effective therapeutic in a mouse model of AD [6,7].

Topical applications of therapeutic peptides, proteins, and nucleic acids, such as plasmid DNA and siRNA, have been increasingly studied because of the medical importance of treating skin diseases, topical vaccination, and improving skin properties [8,9]. However, the effectiveness of topically administered therapeutic nucleic acids is often diminished by low permeation efficiency across the skin due to the pertinent protective physical barriers, including the stratum corneum, nucleated epidermal layers, and tight junctions [10]. Several previous studies have reported that, not only the physical methods, such as electroporation, microneedles, or iontophoresis [11,12,13,14], but also nanocarriers, such as liposome, polymer or carbon nanotube, are capable of penetrating siRNAs in animal skin [15,16,17]. Additionally, one expected method of improving siRNAs permeation efficiency is the use of the cell penetrating peptides, including the arginine-rich transcriptional activator (TAT)-derived peptides [18,19,20,21,22,23]. We also previously reported that the combination of TAT and the tight junction opening AT1002 peptide, widely and effectively accelerates transdermal siRNA delivery [24].

In the present study, we develop a hydrogel formulated with sericin (SC), which contained anti-RelA siRNAs and functional peptides, as a topical application for use against AD. We previously reported the silk protein SC as a useful biocompatible biomaterial for the formation of hydrogels without cross-linkages, as an aqueous gel base [25,26]. Therefore, this versatile property made SC a suitable choice for a biomaterial in our preparation. We then determined the intradermal permeation of fluorescence-labeled siRNA as well as the anti-AD effects of an anti-RelA siRNA preparation in different hydrogel formulations following topical skin application in an AD mouse model.

## 2. Experimental Section

### 2.1. RNAs, Peptides, and Animals

The 6-carboxyfluorescein-aminohexyl (FAM)-siRNA, which served as a fluorescence-labeled siRNA, (FAM-siRelA; antisense, 5ʹ-6-FAM-UACGUACUAUCGCGCGGAUdTdT-3ʹ), anti-RelA siRNA (siRelA; antisense, 5ʹ-AAUGUCUUCUUUCUGCACCdTdT-3ʹ), and negative control siRNA (siControl; antisense, 5ʹ-UACGUACUAUCGCGCGGAUdTdT-3ʹ) were obtained from Cosmo Bio Co., Ltd., (Tokyo, Japan). The TAT peptide (Tat, 12-mer: GlyArgLysLysArgArgGlnArgArgArgCysGly), and AT1002 peptide (AT1002, 8-mer: PheCysIleGlyArgLeuCysGly) were obtained from BEX Co., Ltd., (Tokyo, Japan).

Six-week-old female ICR mice were purchased from SLC (Shizuoka, Japan) and 6-week-old male NC/Nga mice were purchased from Charles River Laboratories Japan, Inc. (Kanagawa, Japan). The mice were housed under controlled conditions of 22–24 °C temperature, 40%–60% humidity, and 12-h light/dark cycle with the light period starting at 8:00 a.m. Food and water were supplied *ad libitum*. All experiments with animals were carried out in accordance with a protocol approved by the Animal Care and Ethics Committee of the Tokyo University of Pharmacy and Life Sciences (Project identification number: School of Pharmacy 10-64, 2012).

### 2.2. Preparation of siRNA Complex and SC Based Hydrogels

The siRNA/Tat complexes with a nitrogen to phosphorous (N/P) ratio of 10 were prepared by mixing both solutions for 30 min at room temperature. The siRNA/Tat + AT1002 were prepared by mixing the AT1002 and siRNA/Tat complex solutions before use. SC Hope (SHC, SC purity 98.5%, Kougensha Co., Ltd., Nagano, Japan) was used as the source of intact SC. Glycerin (GL, Wako Pure Chemicals Industries Ltd., Osaka, Japan) was used as the plasticizer in the SC hydrogel. To prepare the SC hydrogel, 1% SC was dissolved in purified water at 100 °C for 20 min. Because the SC solutions contain some insoluble SC and other particulates, each solution was centrifuged at 1000 *g* for 5 min to remove the insoluble material, and then cooled to room temperature. The 1% SC solution was kept at 5 °C for 1 day. Then, GL was added to the SC hydrogel as a plasticizer (*v*/*v* = 1/1), to improve its tensibility.

### 2.3. FAM-siRNA Distribution in Tape-Stripped Mouse Skin

The 6-week-old male ICR mice used in this study were anesthetized with intraperitoneal pentobarbital (50 mg/kg), and their backs were shaved clean using an electric clipper followed by a cream-based hair remover (Kanebo Cosmetics Inc., Tokyo, Japan). Then, the backs of the mice were tape-stripped 20 times with surgical tape (3M Japan Limited, Tokyo, Japan) and 40 μL of FAM-siRNA containing SC hydrogels (naked FAM-siRNA, FAM-siRNA/Tat, and FAM-siRNA/Tat + AT1002) were applied to the backs of the mice. After 10 h, the mice were euthanized, and then the dermal tissue was washed with phosphate-buffered saline (PBS) and resected in 1-cm^2^ cross-sections of skin tissue at the application site. The skin tissue samples were soaked in Tissue Mount (Shiraimatsu, Osaka, Japan) at 4 °C overnight in the dark, mounted with Tissue Mount in cold acetone, and 20-μm frozen sections were cut using a Cryostat HM550 (Thermo Fisher Scientific Inc., Waltham, WA, USA) and fixed in cold acetone. Slides were washed three times with PBS, and then the tissue sections were with Fluoroment-G. To examine the permeability of the siRNAs in the mouse skin, FAM-siRNA were observed using confocal laser microscopy (FV1000D IX81, Olympus Corporation, Tokyo, Japan).

### 2.4. Topical Induction of AD-Like Skin in Ears of NC/Nga Mice

AD-like skin was induced in the left ears of 6-week-old NC/Nga mice. First, the mice were sensitized by topically applying 2,4-dinitrofluorobenzene (DNFB, Wako Pure Chemical Ind., Ltd.) dissolved in acetone to their footpads and shaved backs on day 0 and 3. After the first sensitization exposure, DNFB dissolved in acetone/olive oil was applied to their left ears on day 7 and 10.

### 2.5. Distribution of FAM-siRNA in AD-Like Mouse Ear Skin

The AD-induced mice were used in this experiment on day 9 after the first sensitization. Then, the ears were not tape-stripped. All the mice were anesthetized with intraperitoneal pentobarbital (50 mg/kg). The FAM-siRNA-containing SC hydrogels (40 μL of SC gel containing naked FAM-siRNA or FAM-siRNA/Tat + AT1002) were applied to both sides of the left ear lobes of the mice. After 10 h, the mice were euthanized, and then the ear lobes were washed with PBS and resected. The tissue samples were then processed identically to the tape-stripped mouse skin in Section 2.3, and the permeability of siRNAs in the ear lobes of the AD mice, was similarly observed using confocal laser microscopy.

### 2.6. Evaluation of AD-Induced Mice Treated with siRelA-Containing SC Hydrogels

The AD-like left ears of mice (*n* = 4 per group) were treated topically with 50 μL of siRelA/Tat + AT1002 without SC hydrogel or SC hydrogels containing naked siRelA, siControl/Tat + AT1002, or siRelA/Tat + AT1002 (siRelA: 5 μg; AT1002: 100 μg) three times a week for 2 weeks. The left ears were covered with olive oil after every application while olive oil alone was applied to the untreated control mice ears. The ear thickness and clinical skin severity score were periodically evaluated throughout the study. The mice were not evaluated blindly. Ear thickness was measured using a digital thickness gauge (Mitutoyo Co., Kanagawa, Japan) and skin lesions were examined macroscopically. The clinical score was determined by assessing the individual clinical severity of the five symptoms (1. Redness, 2. Deformation, 3. Hemorrhage, 4. Dryness, and 5. Thickness) and assigning scores from 0 to 3 (Maximum score: 15). Then, on day 15, 20-μm frozen sections of left ear lobe tissue samples of the AD mice were prepared and stained with hematoxylin and eosin (H&E) or toluidine blue (TB) and stained sections were observed using microscopy.

### 2.7. Statistical Analysis

All values are expressed as means ± standard error (S.E.). The statistical analysis of the data was performed using analysis of variance (ANOVA) followed by Dunnett’s test. Statistical significance was defined as ** *P* < 0.01.

## 3. Results and Discussion

### 3.1. Intradermal siRNA Distribution in Tape-Stripped Back Skin of Mice

**Figure 1:**
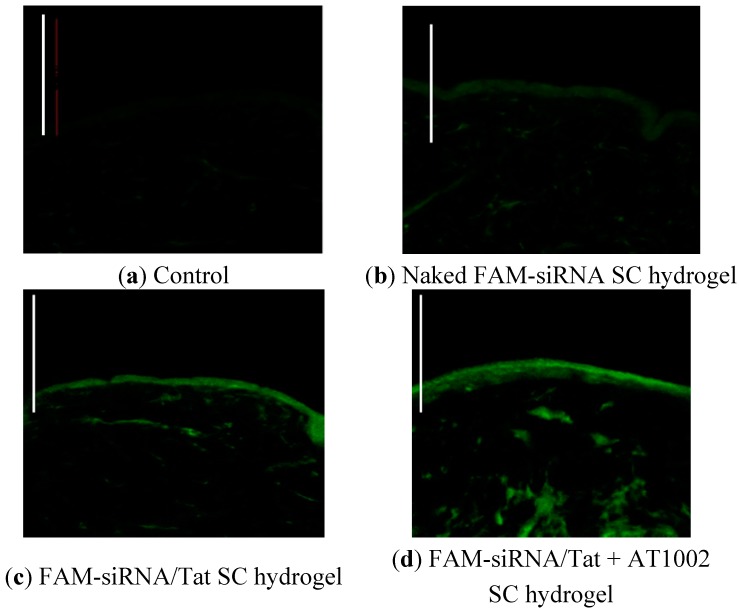
Intradermal distribution of FAM-siRNA in tape-stripped back skin of mice. (**a**) Control; (**b**) Naked FAM-siRNA SC hydrogel; (**c**) FAM-siRNA/Tat SC hydrogel or (**d**) FAM-siRNA/Tat + AT1002 SC hydrogel was applied to 20 times tape-stripped back skin of ICR mice (FAM-siRNA: 5 μg, AT1002: 100 μg). Control refers to non-applied mice. The skin was harvested after 10 h. The frozen skin sections (20 μm) were prepared and observed by confocal laser microscopy. Bar: 100 μm.

The distribution of the FAM-siRNA in the mouse epidermis following the application of SC hydrogels containing naked FAM-siRNA, FAM-siRNA/Tat, or FAM-siRNA/Tat + AT1002 was determined. As shown in Figure 1, FAM-siRNA was not observed in mice treated with naked FAM-siRNA or control mice. In contrast, mice treated with FAM-siRNA containing functional peptides, showed the presence of FAM-siRNA in the skin and the strongest FAM-siRNA fluorescence was observed in the dermis of the mice treated with siRNA/Tat + AT1002. This result suggests that the combination of Tat (cell-penetrating peptide) and AT1002 (tight junction opening peptide) functionally enhanced the delivery of siRNA to deeper sites in the treated mouse skin.

### 3.2. Intradermal siRNA Distribution in AD-Like Mouse Ear Skin

**Figure 2:**
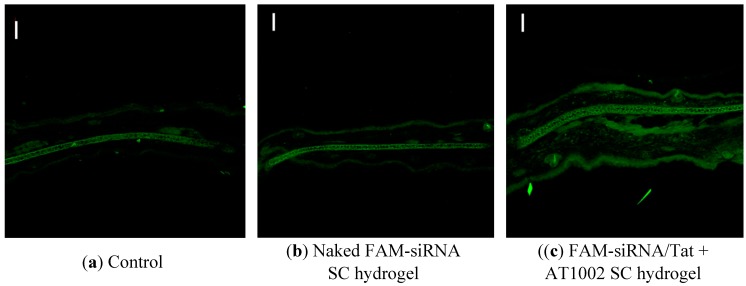
Intradermal distribution of FAM-siRNA in AD-like ear of model mice. (**a**) Control; (**b**) Naked FAMsiRNA SC hydrogel; or (**c**) FAM-siRNA/Tat+AT1002 SC hydrogel was applied to the left ear skin of AD model mice after 10 h. Control refers to non-applied mice. The ear-skin sections (20 μm) were observed by confocal laser microscopy. Bar: 100 μm.

We also examined whether siRNA could penetrate the AD-like ear skin following topical application of the FAM-siRNA/Tat+AT1002 SC hydrogel on day 9 after the first sensitization (Figure 2). No fluorescence was observed in the ears of control mice and those treated with the naked FAM-siRNA containing SC hydrogel, except for autofluorescence of the ear bone. In contrast, mice treated with the FAM-siRNA/Tat+AT1002-containing SC hydrogel, strong FAM-siRNA fluorescence was observed in the ear skin (Figure 2). This result suggests that the hydrogel containing siRelA combined with Tat and AT1002 enhanced the delivery of the siRelA deeper and wider in the AD-like ear skin of mice than the other formulations did.

### 3.3. Effects of Anti-siRelA on Ear Thickness and Clinical Score in NC/Nga AD Mice

**Figure 3:**
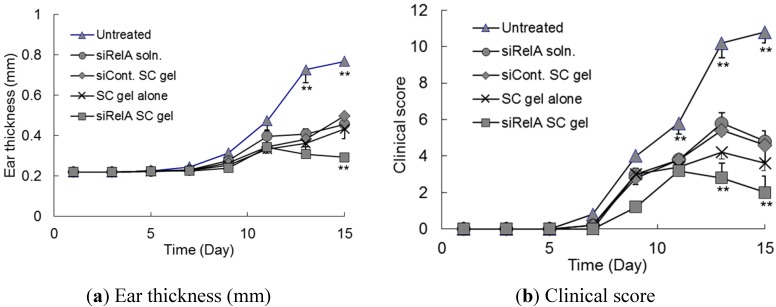
Ear thickness and clinical score assessment of AD-like ear lobes of model mice treated with siRelA containing SC hydrogels. The siRelA/Tat + AT1002 without SC hydrogel, or SC hydrogels containing naked siRelA, siControl (siCont.)/Tat+AT1002 or siRelA/Tat + AT1002 (siRelA: 5 μg, Tat: 32 μg, AT1002: 100 μg) were applied to AD-like left ears of mice three times a week for 2 weeks. (**a**) Ear thickness and (**b**) total clinical score of AD-like left ear of model mice were measured before each application. Each point represents the mean ± S.E. (*n* = 4). ** *P* < 0.01 vs. other groups at the same day.

We examined whether SC hydrogels containing anti-RelA siRNA (siRelA) had a therapeutic effect on the clinical symptoms of AD. Several siRelA formulations were applied three times a week for two weeks from day 3 of the sensitization. The ear thickness and total clinical score of dermatitis in the AD-induced NC/Nga mice are shown in Figure 3. The ear thickness and clinical score of dermatitis gradually increased until day 15 in untreated control mice. In contrast, this effect was strikingly suppressed more in mice treated with the siRelA or SC hydrogels than it was in the untreated control mice (Figure 3). There was no difference in the ear thickness and clinical score of dermatitis between the siRelA/Tat + AT1002 without SC hydrogel, SC hydrogel alone, and SC hydrogel containing siControl/Tat + AT1002 treated groups. However, the dryness score in SC hydrogel-treated groups was more suppressed than that of the mice treated with siRelA/Tat + AT1002 without SC hydrogel. These results indicate that the hydrogel alone suppressed the AD symptom because of its moisturizing effects. In addition, previous studies have reported that SC has not only moisturizing effects but also antioxidant and UV-resistant as well [27,28,29]. Therefore, the SC hydrogel-treated groups showed improvements in AD symptoms, as well as the siRelA without hydrogel-treated groups. Furthermore, the siRelA/Tat + AT1002-containing SC hydrogel showed the lowest values of both ear thickness and clinical scores, indicating that this strong effect could be attributed to the additive effects of the RelA silencing of siRelA/Tat + AT1002 and the moisturizing, antioxidant, and UV-resistant effects of SC hydrogel.

**Figure 4:**
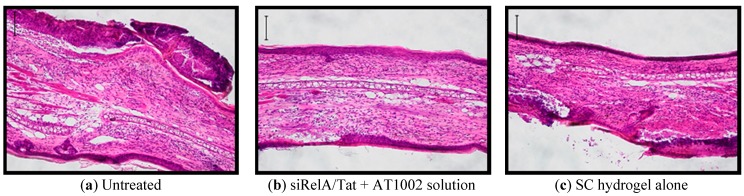

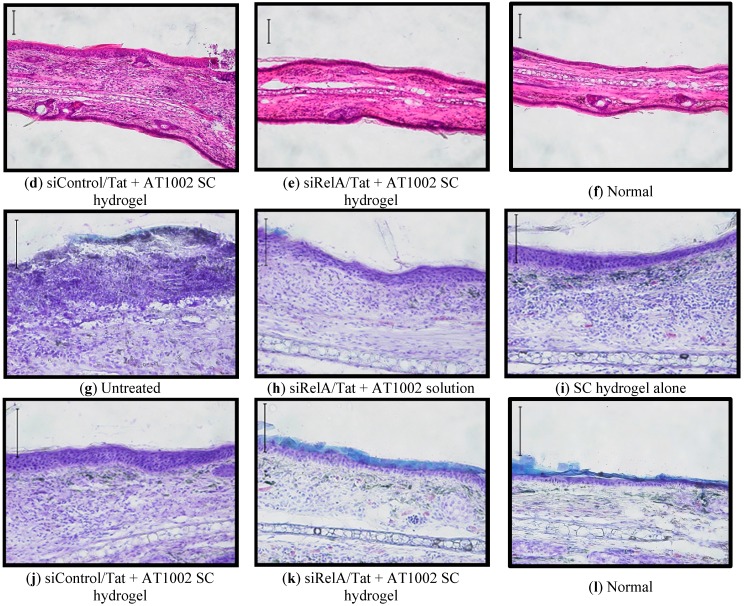
Histological observation of AD-like left ear of model mice after treatment with siRelA containing SC hydrogels. The ear swelling (HE stain, (**a**–**f**)) and infiltration of mast cell (TB stain, (**g**–**l**)) in left ear sections (20 μm) were observed by an optical microscope. Bar: 100 μm.

Finally, the tissue sections of left earlobes of NC/Nga mice were examined histologically with H&E and TB staining on day 15 (Figure 4). H&E staining demonstrated obvious hyperplasia of the epidermis and dermis, and infiltration of eosinophils in the dermis of AD-induced mice. TB staining indicated that mast cells were degranulated in AD-induced mice.

As shown Figure 4a–f, epidermal hyperplasia with areas of parakeratosis and severe infiltrations of eosinophils and a few mononuclear cells in the dermis of ears were highly observed in the untreated mice. In addition, a few mononuclear dermal cells were observed in the empty and siControl-containing SC hydrogel-treated groups; however, none were observed in the siRelA-treated groups. As shown Figure 4a–e, an increase in the number of mast cells was observed and most were degranulated in the skin lesions of untreated mice. However, this was hardly evident with the empty and siControl-containing SC hydrogels-treated groups, and clearly not present in the SC hydrogels containing siRelA-treated group (Figure 4g–l). These results indicate that the SC hydrogel containing siRelA/Tat + AT1002 strongly suppressed symptom of AD in the mouse model because of the additive effects of silencing siRelA by the siRelA/Tat + AT1002, and moisturizing, antioxidant, and UV-resistant effects of the SC hydrogel.

## 4. Conclusions

In the present study, we found that the siRNA was more widely delivered to the site of application in AD-induced ear skin of mice after topical application via the hydrogel with functional peptides than via the preparation without functional peptides. In addition, anti-AD effects in the AD-induced mice treated with hydrogel containing siRelA combined with functional peptides strongly improved by the additive effects of silencing effect by the siRelA/Tat + AT1002, and moisturizing, antioxidant, and UV-resistant effects of the SC hydrogel. In general, the systemic side effects with weakened immune systems by the long-term reduction of NF-κB reduction are important issue. However, the systemic side effects could not be occurred in this study, because siRelA was administered at the just topical inflammatory site. These findings indicate that SC hydrogel containing siRNA/Tat + AT1002 has the potential for application as a versatile and efficacious topical hydrogel formulation, not only for use in AD, but also numerous other skin diseases.
